# *Smilax guianensis* Vitman Extract Prevents LPS-Induced Inflammation by Inhibiting the NF-κB Pathway in RAW 264.7 Cells

**DOI:** 10.4014/jmb.1911.11042

**Published:** 2020-03-24

**Authors:** Ju Gyeong Kim, Min Jeong Kim, Ji Su Lee, Kongmany Sydara, Sangwoo Lee, Sanguine Byun, Sung Keun Jung

**Affiliations:** 1 School of Food Science and Biotechnology, Kyungpook National University, Daegu 4566, Republic of Korea; 2 Division of Bioengineering, Incheon National University, Incheon 01, Republic of Korea; 3 Ministry of Health, Institute of Traditional Medicine, Vientiane 116, Lao PDR; 4 International Biological Material Research Center, Korea Research Institute of Bioscience and Biotechnology, Daejeon 311, Republic of Korea; 5 Department of Biotechnology, Yonsei University, Seoul 03722, Republic of Korea; 6 Institute of Agricultural Science and Technology, Kyungpook National University, Daegu 415, Republic of Korea

**Keywords:** *Smilax guianensis* Vitman extract, nutraceuticals, nitric oxide, nitric oxide synthase, inflammation, NF-κB

## Abstract

Nutraceutical treatments can reduce inflammation and prevent the development of inflammatory diseases. In this study, the anti-inflammatory effects of *Smilax guianensis* Vitman extract (SGE) were examined. SGE suppressed lipopolysaccharide (LPS)-mediated nitrite production in RAW 264.7 cells. SGE also prevented the LPS-induced expression of inducible nitric oxide synthase (iNOS) but not cyclooxygenase (COX)-2. Western blot analysis showed that SGE attenuated LPS-induced phosphorylation of IκB kinase (IKK), inhibitor of kappa B (IκB), and p65. Additionally, SGE inhibited LPS-induced IκB degradation in RAW 264.7 cells. Western blot analysis of the cytosolic and nuclear fractions, as well as immunofluorescence assay results, revealed that SGE suppressed LPS-induced p65 nuclear translocation in RAW 264.7 cells. Moreover, SGE reduced LPS-induced interleukin (IL)- 1β, IL-6, and tumor necrosis factor-α (TNF-α) mRNA expression and IL-1β and IL-6 protein expression in RAW 264.7 cells. Collectively, these results indicate that SGE suppresses the NF-κB signaling pathway and thereby inhibits the production of NO, IL-1β, and IL-6.

## Introduction

The immune system protects against external pathogens such as viruses and bacteria, and toxic chemicals [1]. Unintentional and/or abnormal activation of immune cells (*e.g.* macrophages) can produce excessive inflammatory mediators including pro-inflammatory cytokines, nitric oxide (NO), and reactive oxygen species (ROS) [2]. Therefore, inflammation is a negative consequence of unbalanced immune responses, and it plays a role in cancer, diabetes, and cardiovascular diseases [3, 4]. NO is a free radical formed in immune cells such as macrophages and lymphocytes, and it regulates various physiological and pathophysiological responses including host defense, blood pressure, and platelet function. However, NO produced by macrophages helps mediation of pro-inflammatory and destructive effects [5]. iNOS, the major protein involved in NO production, is closely associated with inflammatory diseases such as atherosclerosis, septic shock, transplant rejection, and neurodegeneration [6]. COX, a prostaglandin H synthase enzyme, is associated with inflammation [7].

The NF (nuclear factor)-κB signaling pathway is regulated by Toll-like receptors and has a key role in inflammation [8]. In the inactive state, NF-κB is a dimer composed of p65 and p100/50, and it is bound to IκB in the cytoplasm. When activated, NF-κB and IκB disassociate, allowing NF-κB to translocate into the nucleus [8, 9]. There, NF-κB transcribes inflammatory genes such as iNOS, COX-2, and inflammatory cytokines (*e.g.*, IL-1β, TNF-α, and IL-6) [10].

Researchers are currently investigating natural products such as plant extracts to find new and potent anti-inflammatory agents that can inhibit NO production and/or NF-κB activation. Throughout history, people have used botanical plants to treat a variety of human diseases. The natural products extracted from medicinal plants are a rich source of biologically active compounds. Some of these compounds have spawned the development of new pharmaceuticals [11]. The genus *Smilax* has more than 350 species and is grown worldwide in tropical and subtropical climates. *Smilax* rhizomes are known to have antibacterial, antifungal, and antioxidant characteristics [12], and they have been used in traditional medicine and beer brewing [13]. *Smilax guianensis* Vitman leaf extracts have antioxidant, antimicrobial, and hemolytic activities in vitro [12]. However, the anti-inflammatory effect of *Smilax guianensis* Vitman remains unclear. Therefore, this study examined the effect of *Smilax guianensis* Vitman extracts (SGE) on LPS-induced NO production and the NF-κB signaling pathway.

## Materials and Methods

### Materials

Dulbecco’s Modified Eagle’s Medium (DMEM), penicillin-streptomycin, and fetal bovine serum (FBS) were obtained from Thermo Scientific HyClone (USA). Lipopolysaccharides from *Escherichia coli* 0111:B4 were obtained from Sigma (USA). The antibody against β-actin was purchased from Santa Cruz Biotech (USA). Antibodies specific for iNOS, COX-2, IKKα, phospho-IKKα/β (Ser176/180), IκBα, phospho-IκBα (Ser32), NF-κB p65, phospho- NF-κB p65 (Ser536), p44/42 (Erk1/2), phospho-p44/42 (Erk1/2) (Thr202/Thr204), SAPK/JNK, phospho-SAPK/JNK (Thr183/Tyr185), p38 MAPK, phospho-p38 MAPK (Thr180/182), and α/β-tubulin were purchased from Cell Signaling Biotechnology (USA). The antibody against Lamin B1 was obtained from Abcam (UK).

### SGE

SGE was purchased from the Korea Research Institute of Bioscience and Biotechnology (Korea). *Smilax guianensis* Vitman was collected in Non Xan village, Xayboury district, Savannakhet province in Laos and identified by Kongmany Sydara of the Institute of Traditional Medicine (Laos) in July 2010. A voucher specimen (accession number KRIB 0033789) of the retained material is preserved at the herbarium of KRIBB. The dried and refined, whole *Smilax guianensis* Vitman plants (91 g) were extracted by placing the plants in 1 L of 99.9% (v/v) methanol and conducting repeating rounds of sonication (15 min) and resting (2 h) for 3 days at 45°C. The resulting product was filtered and concentrated with a rotary evaporator (N-1000SWD; EYELA, Korea) under reduced pressure at 45°C. A total of 5.46 g of methanol-extracted *Smilax guianensis* was obtained by freeze-drying.

### Cell Culture

RAW 264.7 murine macrophage cells were purchased from the Korean Cell Line Bank (Republic of Korea). They were cultured in DMEM containing 10% fetal bovine serum (FBS) supplemented with 1% penicillin-streptomycin at 37°C in a 5% CO_2_ humidified incubator (Thermo Fisher Scientific, USA).

### Cell Viability

Cell viability was analyzed with an MTS assay (Promega, USA) using both 3-(4,5-dimethylthiazol-2-yl)-5-(3-carboxymethoxyphenyl)-2-(4-sulfophenyl)-2H-tetrazolium (MTS) and phenazine methosulfate (PMS).

RAW 264.7 cells were seeded (2 × 10^5^ cells/ml) on a 96-well plate and allowed to reach 70-80% confluency. After incubating for 24 h with various concentrations of SGE (25, 50, and 100 μg/ml), the appropriate amount (20 μl) of the MTS/PMS mixture (Promega) was added, and cell viability was measured with a microplate reader at an absorbance of 490 nm (Bio-Rad Inc., USA).

### Nitrite Assay

RAW 264.7 cells were seeded (2 × 10^5^ cells/ml) on a 96-well plate and allowed to reach 70-80% confluency. The media were substituted with other media containing various concentrations of SGE (25, 50, and 100 μg/ml) and incubated for 1 h. LPS (1 μg/ml) was added, and the cells were incubated for 24 h. To measure the presence of nitrite, the culture media were transferred to a new 96-well plate and mixed with Griess reagent (0.2% N-(1-naphthyl)ethylenediamine and 1% sulfanilamide in 5% phosphoric acid) for 15 min. The absorbance was measured with a microplate reader at 550 nm (Bio-Rad Inc.).

### Western Blot

RAW 264.7 cells were seeded (3 × 10^5^ cells/ml) on a 10 cm dish and allowed to reach 70-80% confluency. The media were then replaced with other media supplemented with various concentrations of SGE (25, 50, and 100 μg/ml). After incubation for 1 h, LPS (1 μg/ml) was added to the media, and the cells were incubated for a specific time period. The media were discarded after incubation. The cells were washed with phosphate-buffered saline (PBS) and then collected with cell lysis buffer (Cell Signaling Biotechnology). After incubating the lysate on ice for 30 min, the supernatant was separated by centrifugation at 13,652 ×*g* for 15 min. The protein concentration was measured by a DC Protein Assay Kit (Bio-Rad Inc.). The cell lysates were mixed with SDS-sample buffer and subjected to 10% SDS-PAGE. The proteins were then transferred to a polyvinylidene fluoride membrane (Millipore, Immobilon-P transfer membrane) and incubated with a specific primary antibody overnight at 4°C. After incubation with the secondary antibody, the protein bands were visualized with a chemiluminescence detection kit (ATTO, Japan) and a Chemiluminescence Systems instrument (SYNGENE, UK).

### Cytosolic and Nuclear Fractions

RAW 264.7 cells were seeded (3 × 10^5^ cells/ml) on a 10 cm dish and allowed to reach 70-80% confluency. The media were then replaced with media containing various concentrations of SGE (25, 50, and 100 μg/ml). After incubation for 1 h, LPS (1 μg/ml) was added to the media, and the cells were incubated for 30 min. The media were discarded after incubation. As per the manufacturer’s instructions, the cells were washed twice with PBS, collected, and separated with the NE-PER Nuclear and Cytoplasmic Extraction Reagent (Thermo Fisher Scientific). Lastly, the extracts were subjected to western blot analysis.

### Immunofluorescence

RAW 264.7 cells were seeded (5 × 10^4^ cells/ml) in an 8-well chamber (ibidi, Germany) and allowed to reach 50-70% confluency. The media were then substituted with media containing varying amounts of SGE (25, 50, and 100 μg/ml). After incubating for 1 h, LPS (1 μg/ml) was added to the media, and the cells were incubated for 5 min. After incubation, the cells were fixed with 4% formaldehyde and permeabilized with ice-cold 100% MeOH. After blocking, cells were incubated with anti-p65 antibody (VECTASHIELD: Vector Laboratories, USA) overnight at 4°C. Goat anti-rabbit IgG H&L conjugated to Alexa Fluora 488 secondary antibodies (Abcam) were incubated with the cells for 1-2 h. The nuclei were counterstained with DAPI. The location of NF-κB p65 was determined using a confocal laser scanning microscope (Carl Zeiss Co. Ltd., Germany).

### Quantitative Real-Time PCR

RAW 264.7 cells were seeded (3 × 10^5^ cells/ml) on 10 cm dishes and allowed to reach 50-60% confluency. The media were then exchanged with other media containing varying amounts of SGE (25, 50, and 100 μg/ml). After incubating for 1 h, LPS (1 μg/ml) was added to the media, and the cells were incubated for 24 h. Total RNA was extracted using an RNA isolation buffer (TaKaRa, Japan) according to the manufacturer’s instructions. The RNA was converted into cDNA by reverse-transcription using the ReverTra Ace qPCR Rt Master Mix (TOYOBO, Japan). The target gene was amplified using specific oligonucleotide primers and a thermal cycler (TaKaRa). The primer sequences are displayed in Table 1. Relative gene expression was determined by real-time PCR with SYBR Green Real-Time PCR Master Mix (TOYOBO) on a real-time PCR detection system (Bio-Rad Inc.) using the comparative ΔΔCq method and the housekeeping gene GAPDH to normalize the data.

### Enzyme-Linked Immunosorbent Assay (ELISA)

RAW 264.7 cells were seeded (1.5 × 10^5^ cells/ml) into a 12-well plate and allowed to reach 70%–80% confluency. The medium was replaced with fresh medium containing various concentrations of SGE (25, 50, and 100 μg/ml) and incubated for 1 h. LPS (1 μg/ml) was added, and the cells were incubated for 24 h. The culture supernatants were collected, centrifuged at 13,000 ×*g* for 10 min to remove particulate matter, and stored at −80°C in fresh tubes. The concentrations of IL-6 and IL-1β in the cell culture supernatants were determined using corresponding ELISA kits (R&D Systems Inc., USA), which were performed according to manufacturer’s instructions. In brief, the collected supernatants were dispensed into a 96-well plate coated with mouse anti-IL-6 or anti-IL-1β capture antibody. The plate was sealed and incubated at room temperature for 2 h. After the addition of anti-IL-6 or anti-IL-1β detection antibody into each well followed by three washes, streptavidin-HRP was added. The wells were washed again, and substrate solution was added. After 20 min, the reaction was terminated using 2N sulfuric acid solution. The absorbance was measured at 450 nm with a reference wavelength of 570 nm using the Varioskan Lux multimode microplate reader (Thermo Fisher Scientific).

### Statistical Analysis

Where appropriate, data are expressed as the mean ± standard deviation (SD), and significant differences between LPS and SGE groups were calculated with Student’s t-test with two-tailed distributions and two-sample equal variance. A probability value of *p* < 0.05 was used as the criterion for statistical significance.

## Results

### SGE Inhibits LPS-Induced Nitrite Production and iNOS Expression in RAW 264.7 Cells

**Fig. 1:**
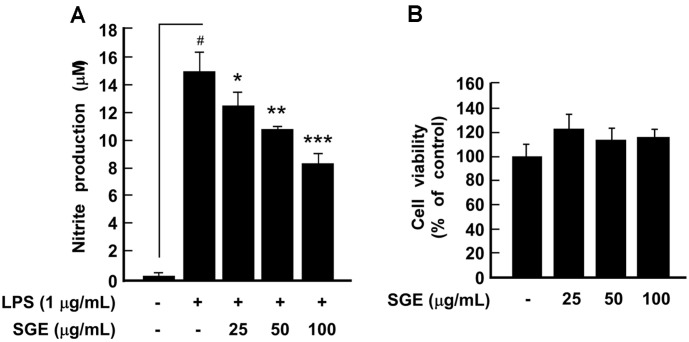
The effect of *Smilax guianensis* Vitman extract (SGE) on lipopolysaccharide (LPS)-induced nitrite production and cell viability in RAW 264.7 cells. (**A**) SGE inhibited LPS-induced nitrite production. Nitrite production was measured using Griess reagent. (**B**) SGE was not cytotoxic to RAW 264.7 cells at concentrations of 25-100 μg/ ml. Cell viability was measured using an MTS assay as described in the Materials and Methods section. The data represent the mean ± SD of three independent experiments. #*p* < 0.05 between control and LPS-exposed cells (no SGE); *p < 0.05, **p < 0.01 and ***p < 0.001.

**Fig. 2:**
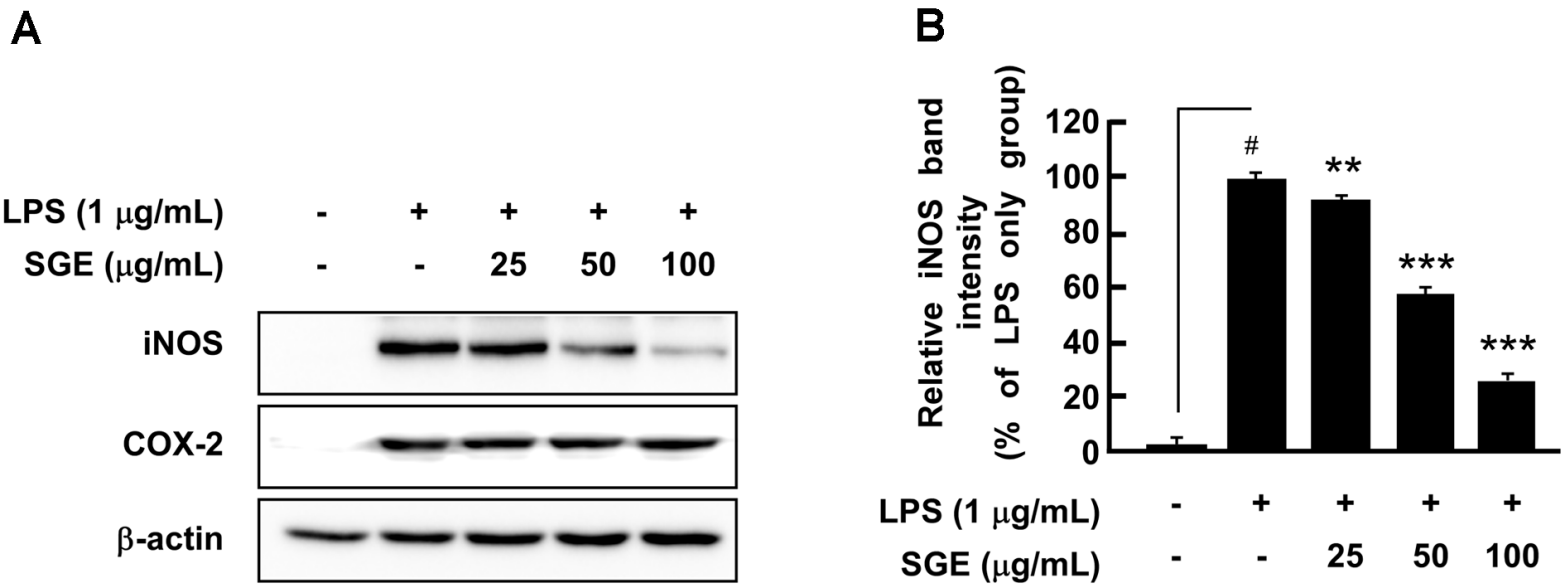
The effect of SGE on iNOS and COX-2 expression in RAW 264.7 cells. SGE inhibited LPS-induced iNOS expression but not COX-2 expression. Cells were pre-treated with SGE for 1 h and then treated with LPS for 24 h. Protein levels were analyzed by western blot analysis. The data represent the mean ± SD of three independent experiments. #*p* < 0.05 between control and LPS-exposed cells (no SGE); **p < 0.01 and ***p < 0.001.

The production of NO plays an important role in pathogen-mediated inflammation [14]. Therefore, we investigated the effect of SGE on LPS-induced NO production in RAW 264.7 cells. SGE significantly suppressed LPS-induced NO production (Fig. 1A). Also, cell viability assays showed that SGE was not toxic to the cells at concentrations of 25-100 μg/ml (Fig. 1B). iNOS and COX-2 are known to mediate inflammation by expressing NO and Prostaglandin E_2_ (PGE_2_), respectively [15]. Western blot analysis showed that SGE suppressed LPS-induced iNOS expression, but it did not affect COX-2 expression (Fig. 2).

### SGE Inhibits LPS-Induced Phosphorylation of IKK, IκB, and p65 in RAW264.7 Cells

**Fig. 3:**
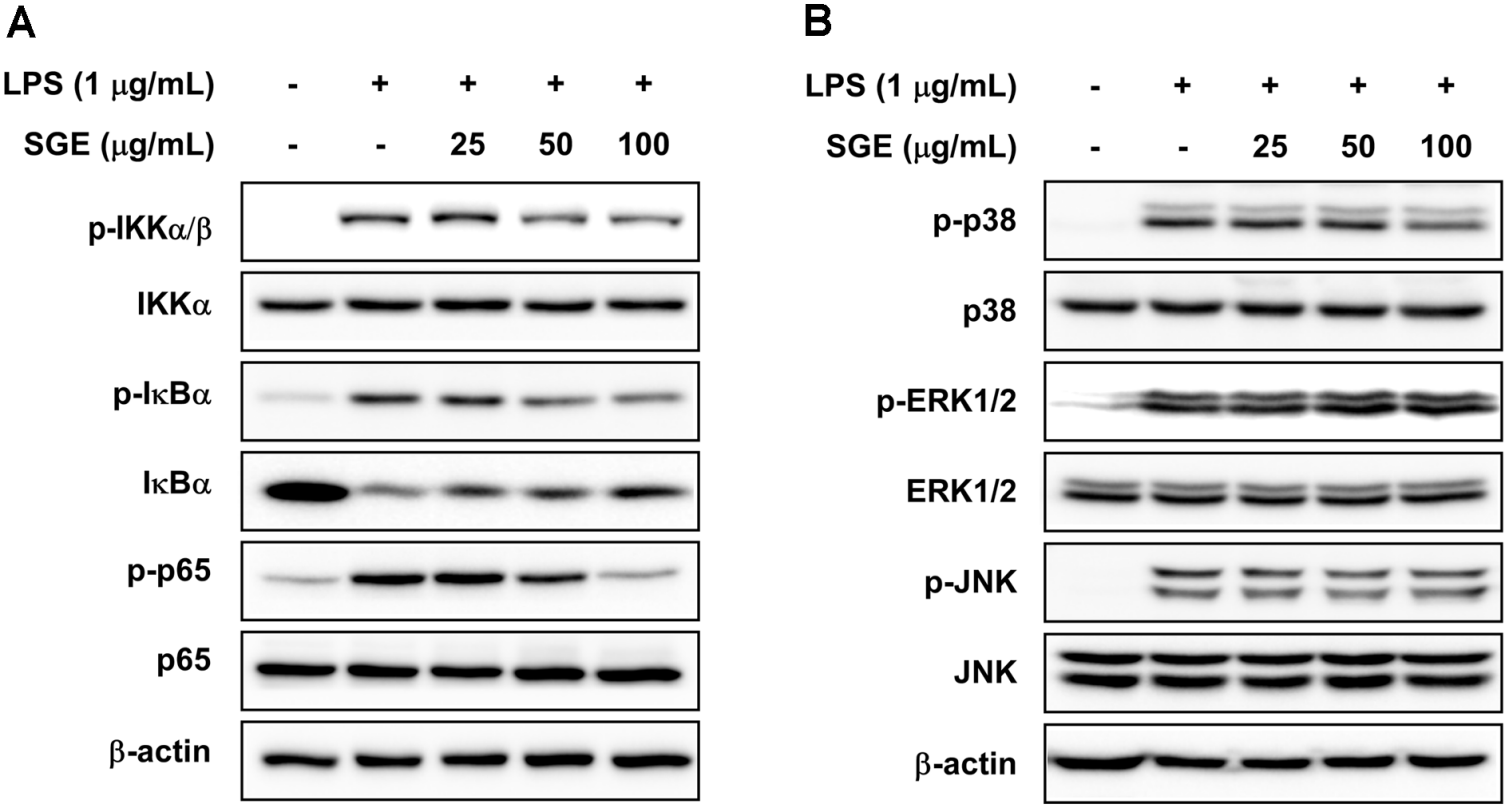
The effects of SGE on LPS-induced NF-κB and MAPK signaling pathways in RAW 264.7 cells. (**A**) SGE inhibited LPS-induced phosphorylation of IKKα/β, IκBα, and p65. (**B**) SGE does not affect LPS-induced phosphorylation of p38, ERK1/2, or JNK. The phosphorylation levels of NF-κB and MAPKs were measured by western blot analysis.

Since iNOS is regulated by NF-κB activity and MAPK phosphorylation [16], we examined the effects of SGE on LPS-induced NF-κB signaling pathway activity and MAPK phosphorylation. SGE suppressed LPS-induced phosphorylation of IKK, IκB, and p65. It also inhibited the degradation of IκB (Fig. 3A). However, SGE did not affect LPS-induced phosphorylation of p38, extracellular-signal-regulated kinase (ERK), or c-Jun N-terminal kinase (JNK) (Fig. 3B).

### SGE Inhibits LPS-Induced Nuclear Translocation of p65 in RAW264.7 Cells

**Fig. 4:**
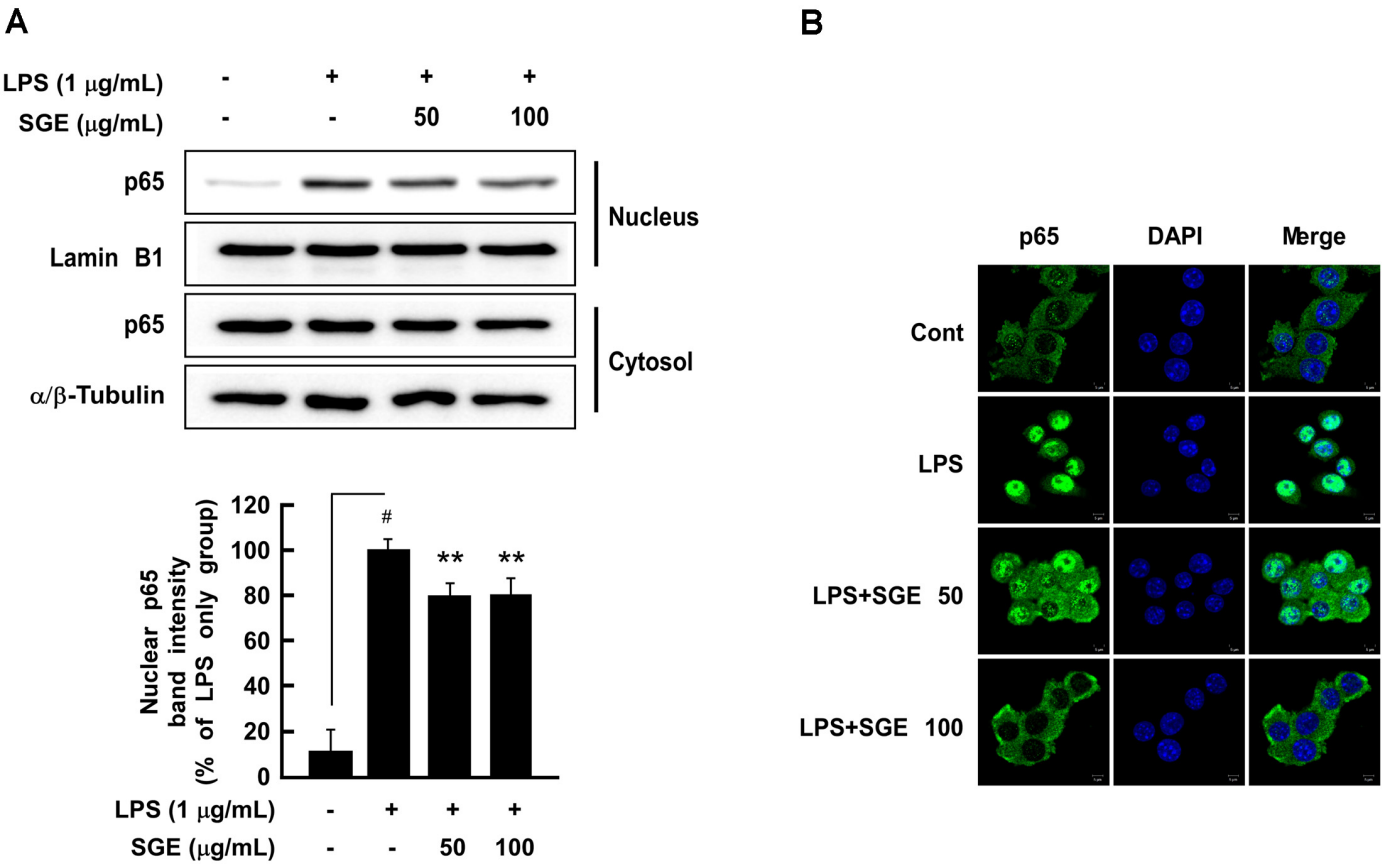
The effect of SGE on LPS-induced nuclear translocation of p65 in RAW 264.7 cells. (**A**) SGE (**B**) inhibited LPS-induced p65 nuclear translocation. p65 was detected using the (**A**) cytosolic and nuclear fractions coupled with (**B**) immunofluorescence as described in the Materials and Methods section. The data represent the mean ± SD of three independent experiments. #*p* < 0.05 between control and LPS-exposed cells (no SGE); **p < 0.01.

Activated NF-κB translocates from the cytosol to the nucleus and subsequently transcribes genes encoding iNOS and COX-2, which induce inflammation [17]. Thus, we assessed the effect of SGE on LPS-induced translocation of the p65 subunit of NF-κB from the cytoplasm to the nucleus in RAW 264.7 cells using western blot and immunofluorescence (Fig. 4). When compared to the LPS-only group, 50 and 100 μg/μl of SGE significantly suppressed the translocation of p65 from the cytoplasm to the nucleus by 78.8% and 79.9%, respectively (Fig. 4A). Furthermore, immunofluorescence analysis showed that SGE suppressed LPS-induced nuclear translocation of p65 (Fig. 4B).

### SGE Inhibits LPS-Induced Pro-Inflammatory Cytokine Expression in RAW264.7 Cells

**Fig. 5:**
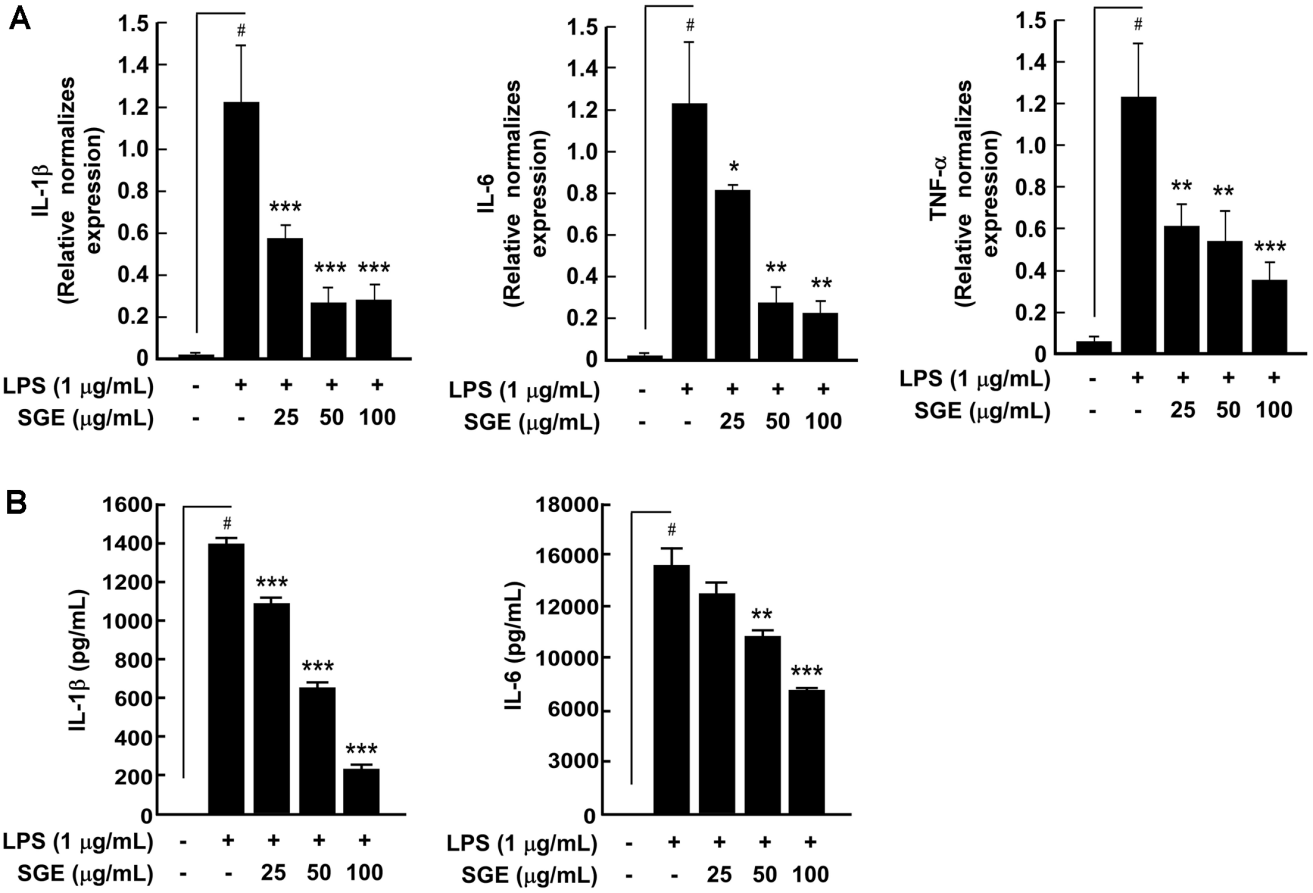
The effect of SGE on LPS-induced pro-inflammatory cytokines in RAW 264.7 cells. SGE suppressed LPSinduced (**A**) expression of IL-1β, IL-6, and TNF-α mRNA and (**B**) production and release of IL-1β and IL-6 proteins. Cells were pre-treated with SGE for 1 h and then treated with LPS for 24 h. IL-1β, IL-6, and TNF-α mRNA and protein levels were measured as described in the Materials and Methods section. The expression levels of pro-inflammatory cytokines were compared via amplification using specific primers. The data represent the mean ± SD of three independent experiments. #*p* < 0.05 between control and LPS-exposed cells (no SGE); *p < 0.05, **p < 0.01 and ***p < 0.001.

Since the expression of pro-inflammatory cytokines such as TNF-α, IL-6, and IL-1β is known to be involved in inflammation [18], we investigated the effect of SGE on the LPS-induced expression of pro-inflammatory cytokines. SGE significantly suppressed the LPS-induced expression of IL-1β, IL-6, and TNF-α mRNAs in RAW264.7 cells (Fig. 5A) and IL-1β and IL-6 protein expression in culture supernatants (Fig. 5B).

## Discussion

Chronic inflammation is involved in diabetes, cardiovascular diseases, and cancer. Although no produced by macrophages aids in fighting pathogens such as viruses, bacteria, fungi, protozoans, and parasites [19], an excess of its production leads to host organ damage via inflammation. Therefore, modulating excess NO may be helpful in regulating the inflammatory response. In a search for anti-inflammatory materials, we screened 100 botanical extracts that reduce NO production in RAW 264.7 cells (data not shown). Among those extracts, SGE had the strongest inhibitory effect on LPS-induced NO production, and it functioned in a concentration-dependent manner.

iNOS and COX-2 are closely linked with inflammation because they produce NO and PGE_2_, respectively [20]. Thus, we examined the effects of SGE on LPS-induced iNOS and COX-2 expression. We found that SGE significantly suppressed LPS-induced expression of iNOS. Multiple studies have reported that iNOS and COX-2 are regulated by similar signaling pathways such as AP-1 and NF-κB [15, 21, 22]. Thus, we expected SGE to affect COX-2 expression as well, but COX-2 expression was not altered by SGE. Extracts from several plants such as *Angelica sinensis*, *Cornus officinalis* Sieb. et Zucc [23], *Acanthopanax sessiliflorum* Seeman, *Daphne genkwa* Sieb. et Zucc., *Dendrobium nobile* Lindl, *Thuja orientalis* L. [24], *Olea europaea* [25], and *Populus deltoides* Leaf [26] can significantly suppress LPS-induced iNOS expression but do not affect COX-2 expression. These results indicate that even if COX-2 is a critical factor in inflammation, inhibition of iNOS is enough to regulate LPS-mediated NO production in RAW 264.7 cells.

NF-κB regulates inflammatory mediators such as COX-2 and iNOS, and it can be activated by various exogenous substances including pathogens and chemicals. Phosphorylation of IκB by IKK activates NF-κB, leading to the rapid ubiquitination and degradation of IκB in proteasomes [27]. After confirming that SGE suppresses LPS-induced phosphorylation of p65 at Ser536, we investigated the effect of SGE on up-stream kinases of NF-κB including IKK, and IκB. We showed that SGE suppressed the phosphorylation of IκB, IKK, and IκB and inhibited IκB protein degradation in RAW 264.7 cells. Most importantly, the phosphorylation of p65 plays a critical role in the nuclear translocation of NF-κB and its subsequent transcriptional activity [28]. Activation of the MAPK signaling pathway by LPS can indirectly up-regulate NF-κB activity and stimulate complex physiological responses [29]. As a result, the p65 subunit of NF-κB is activated and translocates from the cytosol to the nucleus. Our results suggest that SGE influences a regulator of IKK. In another result, 4'-hydroxy wogonin suppressed LPS-induced activation of NF-κB in RAW 264.7 cells because it suppresses phosphorylation of transforming growth factor beta-activated kinase (TAK) 1, which is upstream of IKK [30]. Furthermore, berteroin inhibits the degradation of interleukin-1 receptor-associated kinase (IRAK) 1 as well as TAK1 phosphorylation in LPS-stimulated RAW 264.7 cells. As a result, berteroin inhibits NF-κB activation [31]. Also, thalidomide inhibits LPS-induced activation of the NF-κB signaling pathway and TNF-α production by down-regulating MyD88 expression [32]. Geraniin inhibits LPS-induced ROS/PI3K/Akt-dependent NF-κB activation in RAW 264.7 cells [33]. In addition to NF-κB, ATF-2 and STAT3 may be involved in LPS-induced inflammation in RAW 264.7 cells [34, 35]. However, further studies will be needed to determine whether SGE is involved in the regulation of ATF-2 or STAT3 and what the impact of these mediators might be with respect to the regulation of LPS-induced NO production.

Next, we investigated the nuclear translocation of p65 using the nuclear and cytosolic fractions of cell lysates as well as immunofluorescence. The results showed that SGE inhibited the nuclear translocation of p65. Because SGE inhibits NF-κB signaling, it suppresses the nuclear translocation of p65 and reduces iNOS expression as well as its NO product.

Pro-inflammatory cytokines including IL-6, IL-1β, and TNF-α are secreted from macrophages after LPS treatment, and they are linked to various chronic diseases, including rheumatoid arthritis, type II diabetes, and cancer [36, 37]. IL-1 family cytokines are closely associated with innate immunity and may also promote autoinflammatory diseases [38]. IL-6 also plays an important role in promoting the acquired immune response by stimulating antibody production and effector T cell development [39]. TNF-α is produced by macrophages, monocytes, T cells, and adipocytes and has important proinflammatory properties that promote immunity, cell proliferation, and apoptosis [40]. The excessive production of these cytokines can induce the overexpression of the iNOS gene, which is closely linked to inflammatory diseases [41]. Therefore, the inhibition of these pro-inflammatory cytokines may be helpful in preventing inflammation. We also observed that SGE inhibited the expression of IL-6, IL-1β, and TNF-α mRNA as well as the production and secretion of IL-6 and IL-1β proteins in response to LPS stimulation.

In this study, we demonstrated the anti-inflammatory effects of SGE caused by its inhibition of NF-κB. This resulted in a decrease in the production of NO, TNF-α, IL-6, and IL-1β cytokines. Overall, our results reveal the potential for SGE as a natural therapy to prevent inflammation.

## Figures and Tables

**Table 1:** The nucleic acid sequences of the primers used for quantitative real-time polymerase chain reaction

Target	Orientation	Sequence (5’ to 3’)
IL-1β	Forward	AGT TGA CGG ACC CCA AAA GAT
	Reverse	GTT GAT GTG CTG CTG CGA GA
IL-6	Forward	TGG GAC TGA TGC TGG TGA CAA C
	Reverse	AGC CTC CGA CTT GTG AAG TGG T
TNF-α	Forward	TGG AAC TGG CAG AAG AGG CAC T
	Reverse	AGA GGC TGA GAC ATA GGC ACC G
GAPDH	Forward	ACT CCA CGA CAT ACT CAG C
	Reverse	TCA ACG GCA CAG TCA AGG
